# SIVA-1 regulates apoptosis and synaptic function by modulating XIAP interaction with the death receptor antagonist FAIM-L

**DOI:** 10.1038/s41419-020-2282-x

**Published:** 2020-02-03

**Authors:** Elena Coccia, Laura Planells-Ferrer, Raquel Badillos-Rodríguez, Marta Pascual, Miguel F. Segura, Rita Fernández-Hernández, Joaquin López-Soriano, Eloi Garí, Eduardo Soriano, Bruna Barneda-Zahonero, Rana S. Moubarak, M. Jose Pérez-García, Joan X. Comella

**Affiliations:** 1 Cell Signaling and Apoptosis Group, Vall d’Hebron Research Institute (VHIR), 08035 Barcelona, Spain; 2 Centro de Investigación Biomédica en Red sobre Enfermedades Neurodegenerativas (CIBERNED), ISCIII, 28031 Madrid, Spain; 3 Institut de Neurociències, Departament de Bioquímica i Biologia Molecular, Facultat de Medicina, Universitat Autònoma de Barcelona, 08031 Bellaterra, Spain; 4 Institut de Neurociències, Universitat de Barcelona, Bellaterra, Spain; 5 Department of Cell Biology, Physiology and Immunology, Institut de Neurociències, Universitat de Barcelona, 08031 Barcelona, Spain; 6 Group of Translational Research in Child and Adolescent Cancer, Vall d’Hebron Research Institute (VHIR)-UAB, 08035 Barcelona, Spain; 7 Cell Cycle Laboratory, Institut de Recerca Biomèdica de Lleida (IRBLleida), and Departament de Ciències Mèdiques Bàsiques; Facultat de Medicina, Universitat de Lleida, 25198 Lleida, Catalonia Spain; 8 ICREA Academia, Barcelona, Spain; 9 Present Address: Department of Pathology, NYU Langone Health, New York, 10016 NY USA

**Keywords:** Cell death in the nervous system, Molecular neuroscience

## Abstract

The long isoform of Fas apoptosis inhibitory molecule (FAIM-L) is a neuron-specific death receptor antagonist that modulates apoptotic cell death and mechanisms of neuronal plasticity. FAIM-L exerts its antiapoptotic action by binding to X-linked inhibitor of apoptosis protein (XIAP), an inhibitor of caspases, which are the main effectors of apoptosis. XIAP levels are regulated by the ubiquitin-proteasome pathway. FAIM-L interaction with XIAP prevents the ubiquitination and degradation of the latter, thereby allowing it to inhibit caspase activation. This interaction also modulates non-apoptotic functions of caspases, such as the endocytosis of AMPA receptor (AMPAR) in hippocampal long-term depression (LTD). The molecular mechanism of action exerted by FAIM-L is unclear since the consensus binding motifs are still unknown. Here, we performed a two-hybrid screening to discover novel FAIM-L-interacting proteins. We found a functional interaction of SIVA-1 with FAIM-L. SIVA-1 is a proapoptotic protein that has the capacity to interact with XIAP. We describe how SIVA-1 regulates FAIM-L function by disrupting the interaction of FAIM-L with XIAP, thereby promoting XIAP ubiquitination, caspase-3 activation and neuronal death. Furthermore, we report that SIVA-1 plays a role in receptor internalization in synapses. SIVA-1 is upregulated upon chemical LTD induction, and it modulates AMPAR internalization via non-apoptotic activation of caspases. In summary, our findings uncover SIVA-1 as new functional partner of FAIM-L and demonstrate its role as a regulator of caspase activity in synaptic function.

## Introduction

Apoptosis plays a crucial role during neural development and adult life^1,2^. The main orchestrators of apoptosis are caspases, effector proteases that mediate the proteolytic cascade that ultimately leads to cell death. Several non-apoptotic functions of caspases have been reported in neurons^2^. Localized activation of caspases has been described as necessary in the physiological context of neuronal pruning, axon guidance, and synaptic plasticity^3^. The activation of caspase-3 via mitochondria is required for AMPA receptor (AMPAR) internalization in NMDA receptor-dependent long-term depression (LTD). Several studies have shown that LTD is abolished in caspase-3 knock-out mice^4^, and caspase-3-deficient neurons in culture fail to show spine shrinkage in response to NMDA stimulation (chemical LTD)^5^.

FAIM-L is the neuron-specific isoform of FAIM, and it harbors 22 additional amino acids at the N-terminal as compared to FAIM-S. We have previously shown that FAIM-L regulates caspase activation in neurons. Moreover, decreased FAIM-L levels have been associated with the progression of Alzheimer’s disease (AD), an irreversible and progressive neurodegenerative disorder that is the most common cause of dementia^6^. AD is characterized by neuronal death and the dysregulation of synaptic plasticity^7^, mechanisms in which FAIM-L and apoptotic pathways are involved and have been shown to play crucial roles.

FAIM-L modulates death receptor-induced cell death by interacting with death receptors^8^ and by interacting and stabilizing the X-linked inhibitor of apoptosis protein (XIAP). XIAP is a key member of the inhibitor of apoptosis protein family that directly binds to and inhibits effector caspases. XIAP levels are regulated by a process of autoubiquitination and proteasome degradation. Given its anti-apoptotic role, modification of XIAP levels is a decisive step in cell death induction^9^. FAIM-L confers neurons additional protection against cell death. In fact, FAIM-L interacts with XIAP and inhibits its ubiquitination and degradation by the proteasome, therefore maintaining the role of XIAP in preventing the cleavage and activation of caspases^10^. FAIM-L also participates in processes where caspases play a non-apoptotic role. By stabilizing XIAP levels, FAIM-L prevents AMPAR internalization after chemical induction of LTD (chLTD) in vitro and protects axons from degeneration induced by growth-factor deprivation^11^.

Structural prediction algorithms for FAIM-L do not identify any functional domain that could predict potential protein association, and apart from XIAP, no other FAIM-L-interacting protein has been described to date. Here, using a yeast two-hybrid screening approach, we screened for potential FAIM-L partners of relevance for its function. Using either the full-length or the specific N-terminal amino acid sequence of FAIM-L as bait, we identified the proapoptotic SIVA-1 protein as a FAIM-L binding partner. SIVA-1 has been described as a proapoptotic protein, capable of inducing cell death in several cellular models^12^. Moreover, SIVA-1 has been reported to interact with XIAP^13^. Apoptotic induction mediated by SIVA-1 has been proposed to occur through several pathways, and it is essential for p53-induced apoptosis in granular neurons^14^. Here, we studied the contribution of SIVA-1 to the roles of FAIM-L and XIAP in caspase-dependent apoptotic and non-apoptotic functions in neurons. We found that SIVA-1 blocks the antiapoptotic function of FAIM-L by displacing the XIAP/FAIM-L interaction, thereby inducing XIAP degradation, caspase-3 activation and subsequent neuronal death or AMPAR internalization. Our results point to SIVA-1 as one of the proteins of the apoptotic machinery able to modulate caspase-3 activation in response to synaptic plasticity. On the basis of our findings, SIVA-1 emerges as a novel synaptic modulator.

## Material and methods

### Reagents

Unless otherwise specified, all biochemical reagents were purchased from Sigma-Aldrich.

### Yeast two-hybrid screen

The pACT2-mouse cDNA library in *Escherichia coli* (Clontech cat# ML4008AH/cat# 638841) was pre-transformed in the yeast AH109 strain (more than 10^7^ independent clones). The full-length FAIM-L and the 22 additional amino acids at the N-terminal FAIM isoform (FAIM-L) bait proteins were subcloned into pGBKT7 vector and transformed in Y187 yeast strain. The two-hybrid selection was performed by mating, following the matchmaker two-hybrid system 3 protocol (cat# K1612-1 Clontech). Positive colonies were selected in drop out medium lacking leucine, tryptophan, and histidine and containing 20 mM aminotriazole. Colonies were analyzed by polymerase chain reaction (PCR). cDNA was sequenced and transformed in *E. coli* (WB Cat# OP50), and interactions of bait and prey were confirmed by back transformation in yeast.

### Cell culture

HEK293T cells (ATCC Cat# CRL-3216) were grown in DMEM supplemented with 10% heat-inactivated fetal bovine serum (iFBS) (Invitrogen), 20 U/ml penicillin and 20 µg/ml streptomycin. Rat pheochromocytoma PC12 cells (ATCC Cat# CRL-1721) were grown in DMEM supplemented with 6% iFBS, 6% heat inactivated horse serum (iHS), 10 mM HEPES, 20 U/ml penicillin and 20 µg/ml streptomycin. Cultures were maintained at 37 °C in a 5% CO_2_ atmosphere in a humidified incubator.

### Primary neuron cultures

Neuron cultures were prepared from wild-type C57BL/6J mice (Envigo, France) at embryonic day 15–16 (E15–16). Cerebral cortices and hippocampi were dissected in phosphate-buffered saline (PBS) pH 7.4. After trypsin and DNase treatment, tissues were mechanically dissociated and filtered through a 40-µm nylon mesh. Cells were resuspended in DMEM supplemented with 5% iFBS, 5% iHBS, 20 U/ml penicillin and 20 µg/ml streptomycin. Cells were then plated in poly-D-lysine-coated plates at a density of 3 × 10^5^ cells/ml or on coverslips at 1.5 × 10^5^ cells/ml for immunocytochemistry experiments. Four hour after seeding, medium was replaced by Neurobasal medium supplemented with B27, glutaMAX (Life Tech), 20 U/ml penicillin and 20 µg/ml streptomycin. Culture medium was partially replaced every 3–4 days with fresh medium. Cultures were kept at 37 °C in a 5% CO_2_ atmosphere in a humidified incubator. When pan-caspase inhibitor quinolyl-Val-Asp-OPh (Q-VD) treatment was performed, Q-VD was added directly to culture media at a final concentration of 10 µM. All experimental protocols were approved by the Vall d’Hebron Institutional Review Board.

### Plasmids

The constructions used for this study, namely 3xHA-SIVA-1, 3xHA-ΔSIVA-1, 3xFLAG-FAIM-L, 3xHA-FAIM-L, 6xMyc-XIAP and YFP, were expressed under the control of a cytomegalovirus constitutive promoter in the pcDNA3 expression vector (Invitrogen). 3xFLAG-SIVA-1 plasmid was kindly provided by Dr. Ulrike Resch (Medical University of Vienna). Lentiviral plasmids for this study were cloned into pEIGW. Short hairpin RNA (shRNA) targeting SIVA-1 was cloned into the pLVTHM vector, and a scrambled sequence was used as a control.

### Lentiviral production

Lentiviruses were produced as described previously by Segura et al.^8^. For infection, lentiviruses were added to the host cell medium. Infection efficiency was monitored by counting green fluorescent protein (GFP)-positive cells.

### Cell transfection and infection

HEK293T (ATCC Cat# CRL-3216) or PC12 (ATCC Cat# CRL-1721) cells were transfected with the desired expression plasmid using the calcium phosphate method or Lipofectamine 2000 (Invitrogen), following the manufacturer’s instructions. The total amount of transfected DNA was kept constant by adding empty pcDNA3 expression vector. Primary neurons were transfected with Lipofectamine 2000, as described in Dalby et al.^15^.

### Immunoprecipitation

After 24–48 h of transient transfection for ectopic expression, or after 24 h in culture, HEK293T and PC12 cells were rinsed in PBS 1× and lysed in immunoprecipitation lysis buffer (IP lysis buffer) containing 20 mM Tris/HCl, pH 7.4, 150 mM NaCl, 2 mM EDTA, 10% Glycerol, 1% Triton X-100, and supplemented with a protease inhibitor cocktail (Roche). Samples were lysed for 30 min on ice and centrifuged at 4 °C at 12,000 × *g*. One milligram of total protein was used for immunoprecipitation overnight at 4 °C in an orbital shaker. Specific FLAG M2 Affinity monoclonal agarose beads or anti-ubiquitin-conjugated agarose beads (Santa Cruz Biotechnology Cat# sc-8017 AC) were used for IP:FLAG and IP:Ubiquitin respectively. For IP:Myc, lysates were precleared by 30 min of incubation with 20 µl conjugated protein G suspension and 0.25 µg/µl control mouse IgG. Afterwards, 1 µg Myc antibody (Santa Cruz Biotechnology Cat# sc-40) was added to each sample and incubated for 4 h at 4 °C in an orbital shaker. Finally, 20 µl conjugated protein G suspension was added and incubated overnight on an orbital shaker. After incubation, agarose beads were washed five times with IP lysis buffer and eluted. Elution was performed with a competitor peptide for IP:FLAG and IP:Myc, following the manufacturer’s protocol. For IP:Ubiquitin, elution was performed by adding Laemmli buffer (60 mM Tris/HCL 1 M, pH 6.8, 2% sodium dodecyl sulfate (SDS), 10% Glycerol, 0.01% Bromophenol Blue) without dithiothreitol and boiling samples at 95 °C for 5 min. Whole-cell lysate and immunoprecipitated proteins were then analyzed by western blot.

### Immunohistochemistry

E16, P0, P5, P15, and adult OF-1 mice (Charles River, Lyon; France) were used for immunohistochemistry experiments. The day the vaginal plug was detected was considered embryonic day 0 (E0), and the day of birth as postnatal day 0 (P0). First, the animals were deeply anesthetized with a Ketolar (Parke-Davies/Pfizer New York, NY; USA)/Rompun (Bayer AG, Leverkusen; Germany) mixture and perfused with 4% paraformaldehyde in 0.1 M phosphate buffer. Brains were dissected, cryoprotected and frozen. Next, 30–50 μm coronal sections were obtained. After blocking, sections were incubated overnight with specific rabbit antibody against SIVA-1 (dilution 1:100, Santa Cruz Biotechnology Cat# sc-48768) or mouse anti-SIVA-1 (dilution 1:100, Sigma-Aldrich Cat#SAB1400393). These primary antibodies were visualized by sequential incubation with biotinylated secondary antibodies (dilution 1:200, Vector Labs Burlingame, CA; USA) and the streptavidin–peroxidase complex (dilution 1:400, Amersham Biosciences Pittsburgh, PA, USA). The peroxidase reaction was developed with diaminobenzidine (DAB) and H_2_O_2_. The sections were mounted onto gelatinized slides, and then dehydrated and cover-slipped with Eukkit (Panreac).

Sections from E16, P5, P15, and adult mice were used for double immunofluorescence studies. Sections were incubated overnight with the specific antibody detecting SIVA-1 (dilution 1:100, Santa Cruz Biotechnology, Cat# sc-48768) combined with antibodies against neuron-specific nuclear protein (NeuN, dilution 1:100, Millipore Cat# MAB377), PV (dilution 1:3000, Swant Antibodies Cat# PVG-214, RRID:AB_2313848) or GFAP (dilution 1:500, Millipore Cat# AB5804). Primary antibodies were visualized using secondary Alexa Fluor-conjugated antibodies. Sections were mounted with Prolong diamond mounting medium (Thermo Fisher) and viewed under a confocal microscope.

### Immunocytochemistry

Hippocampal or cortical primary neurons were cultured on glass coverslips pre-coated with 10 µg/ml poly-D-lysine. After 7–10 days in vitro (DIV), cells were fixed with 4% paraformaldehyde for 30 min at room temperature. Permeabilization and blocking were carried out with a permeabilization solution (PS) containing 5% FBS, 5% bovine serum albumin, and 0.1% Triton-X for 1 h at room temperature, followed by incubation with primary antibodies in PS overnight at 4 °C. The following primary antibodies were used: anti-SIVA-1 (dilution 1:500 Santa Cruz Biotechnology Cat# sc-48768; Sigma-Aldrich Cat# SAB1400393); anti-SIVA-1 (dilution 1:25, Sigma-Aldrich Cat# SAB3500697), anti-XIAP (dilution 1:25, BD Cat# 610762), anti-HA (dilution 1:100, Roche Cat# 1-867-423); anti-Rab5 (dilution 1:500, Cell Signaling Technology Cat# 2143); anti-Calnexin (dilution 1:500, Cell Signaling Technology Cat# 2433S); anti-βIII Tubulin (dilution 1:1000, Covance Research Products Inc Cat# SIG-3840-1000); anti-TrkA (dilution 1:1000, in house); anti-PSD95 (dilution 1:200, Cell Signaling Cat# 3450); anti-Synapsin II (dilution 1:200, Enzo Life Science Cat# ADI-VAS-SV061-E); and anti-GFP (1:2000, Abcam Cat# ab6556). Subsequently, cells were rinsed with PBS and incubated with fluorescent-conjugated secondary antibodies (dilution 1:400–1:1000, AlexaFluor Thermo Fisher Scientific Cat# A-11001 Cat# A-11912) diluted in PS for 1 h at room temperature. Prior to the last wash, cells were incubated for 30 min at room temperature with Hoechst 33258 (0.05 µg/ml) to stain nuclear DNA. MitoTracker staining (Invitrogen) was used, following the manufacturer’s instructions. Coverslips were finally mounted on slides with Prolong diamond mounting medium (Thermo Fisher).

### Cell death quantification

At 4 days post infection, primary neurons were fixed with 2% paraformaldehyde, permeabilized with 0.1% Triton™ X-100 and stained with Hoechst 33342 (0.05 µg/ml). Apoptosis was assessed by counting viable and dead cells, discriminating condensed and fragmented nuclei (apoptotic nuclear morphology type II)^16^. For each experiment quantification was performed in blind testing, and at least 500 cells were counted per condition.

### Caspase activity assay

The caspase activity assay was performed as previously described by Galenkamp et al.^17^. Briefly, primary neurons were harvested and lysed in caspase activity buffer (20 mM HEPES-NaOH, pH7.2, 10% sucrose, 150 mM NaCl, 5 mM EDTA, 1% Igepal CA-630, 0.1% CHAPS, and 1× EDTA-free complete protease inhibitor cocktail). Next, 20 µg protein was incubated at 37 °C in caspase activity buffer supplemented with 10 mM DTT and 50 µM of the fluorogenic substrate Ac-DEVD-Afc (Merck Millipore). Plates were read in a fluorometer using excitation and emission wavelengths of 405 and 535 nm, respectively.

### Subcellular protein fractionation

Subcellular fractionation was performed as previously described^18^. Adult mouse brains were homogenized in buffer containing 10 mm HEPES, pH 7.4, 2 mm EDTA, 0.32 M sucrose, and protease inhibitor cocktail (Roche). Cell homogenates were centrifuged sequentially at 600 × *g* for 10 min to remove nuclei (N) and unbroken cells, then at 3000 × *g* for 10 min to pellet the plasma membrane (PM) and collect cytosolic fractions (supernatant). Nuclei were extracted from the 600 × *g* pellet with centrifugations at 500 × *g* for 15 min. The supernatants were centrifuged twice at each speed, and pellets were washed twice by resuspension in homogenization buffer and recentrifugation. Pellets were lysed in SET buffer (10 mM Tris-HCl pH7.4, 1 mM EDTA, 150 mM NaCl, 1% SDS). Protein concentration was quantified by a modified Lowry assay (DC protein assay; Bio-Rad, Hercules, CA). Samples were heat-denatured in Laemmli buffer and subjected to SDS polyacrylamide gel electrophoresis.

### Western blot

Cells were harvested and lysed in SET buffer. Protein concentration was quantified by a modified Lowry assay (DC protein assay; Bio-Rad, Hercules, CA, USA). Lysates were prepared with Laemmli buffer (60 mM Tris/HCl 1 M pH 6.8, 2% SDS, 100 mM glycerol, 0.01% Bromophenol Blue), resolved in SDS polycramide gels, and transferred onto polyvinylidene fluoride Immobilon-P membranes (Merck Millipore). After blocking with TBS 1× −0.1% Tween-20 containing 5% nonfat dry milk for 1 h at room temperature, membranes were probed with the appropriate primary antibodies, prior to incubation for 1 h with the appropriate specific peroxidase-conjugated secondary antibody. Membranes were developed using the EZ-ECL chemiluminescence detection kit (Biological Industries, Kibbutz Beit Haemek, Israel). The following primary antibodies were used: anti-FAIM-L (dilution 1:2000, in house); anti-caspase-3 (dilution 1:1000, Cell Signaling Technologies Cat# 9662); anti-pan ERK (dilution 1:10000, BD Bioscience Cat# 610123); anti-β-actin (dilution 1:2000, Santa Cruz Biotechnology Cat# sc-47778); anti-alpha-tubulin (dilution 1:50000, Sigma-Aldrich Cat# T5168); anti-XIAP (dilution 1:5000, BD Bioscience Cat# 610762); anti-FLAG (dilution 1:10000, Sigma-Aldrich Cat# F3165); anti-HA (dilution 1:5000, Roche Cat# 11867423001); anti-SIVA-1 (dilution 1:500, Sigma Cat# SAB3500697); anti-GluA2 (1:1000, Millipore Cat# MAB397); anti-cytochromec (1:1000, BDbioscience Cat# 556433); anti-calnexin (1:1000, Abcam Cat# ab31290); anti-histone-3 (1:1000, Cell Signaling Technologies Cat# 9715); and anti-GAPDH (1:2000, Abcan Cat# ab9485).

### Chemical LTD induction

Primary neurons were treated at 12–14 DIV with 50 µM N-methyl-D-aspartate (NMDA) for 15 min at 37 °C to induce chLTD as described in Li et al.^4^. Fifty micromolar of BAPTA-AM was used as calcium chelator and 1 µg/ml of cycloheximide (CHX) as an inhibitor of protein translation. Cells were pretreated with BAPTA-AM or CHX during 30 or 60 min, respectively. Treatments were added to media for the pretreatment and maintained during chLTD induction.

### GluA2 internalization assay and surface staining

Internalization assays were performed as described in Martinez-Marmol et al.^11^, with minor modifications. Hippocampal neurons at 12–14 DIV were incubated with antibodies against the N-terminus of GluA2 (2 µg/ml, mouse monoclonal, clone 6C4, Millipore Cat# MAB397) for 60 min at 20 °C. They were then either stimulated with medium containing NMDA (50 µM) for 15 min at 37 °C or left unstimulated. Subsequently, they were fixed for 5 min at room temperature in paraformaldehyde:sucrose in PBS. Surface-remaining antibody-labeled receptors were visualized by means of a 1 h incubation with saturated AlexaFluor-647 secondary antibody (15 µg/ml, Abcam Cat# ab150115). Neurons were then permeabilized for 2 min with methanol (−20 °C), and internalized antibody-labeled GluA2 was detected by a 1 h incubation with Alexa 568-conjugated secondary antibody (1 µg/ml; Molecular Probes Cat# A-11004). Simultaneously, infected GFP-positive or HA-positive neurons were stained with antibodies against GFP (5 µg/ml, Abcam; Cat# ab6556) or HA (10 µg/ml Roche Cat# 11867423001), respectively. Thus, the GFP fluorescence of infected neurons was enhanced by a 30 min incubation with Alexa 488-conjugated secondary antibody (4 µg/ml; Thermo Fisher Scientific Cat# A27034). After three washes in PBS 1×, the coverslips were mounted on slides with Prolong diamond mounting medium (Thermo Fisher).

### Image acquisition and analysis

Images were acquired with a confocal laser scanning microscope (spectral FV1000; Olympus). The digital images were processed using the FIJI software (Fiji)^19^.

Protein co-localization in the dual-color confocal images was measured quantitatively using the JACoP Plug-in (http://fiji.sc/wiki/index.php/Colocalization_Analysis). Co-localization was determined by Pearson’s and Manders co-localization coefficients. Briefly, Pearson’s coefficient values describe the relationship between two image signals calculated by linear regression. Values can range from 1 to −1, with 1 standing for complete positive correlation and −1 for complete negative correlation. Manders co-localization coefficients (M_1_ and M_2_) describe the contribution of two selected channels to the pixels of interest, in this case Channel 1 being SIVA-1 and Channel 2 the other markers. Each coefficient represents the fraction of one channel signal that coincides with the signal of the other channel. Manders co-localization coefficients values range from 0 (uncorrelated distributions of two probes with one another) to 1 (perfect co-localization of two images)^20^. At least 15 cells were considered for quantification.

For GluA2 internalization and surface staining, a z-stack of images was obtained through various filter channels (Alexa-488, Alexa-568 and Alexa-674). Typically, 25 serial 2D images were recorded at 45-nm intervals. Image acquisition settings were identical in each experiment. FIJI software was used to make 2D projections from the z-stack of images. We measured the total integrated intensity of internalized GluA2 and surface-remaining GluA2 in the same region of infected (GFP/HA-positive) neurons. For each experiment at least 15–20 cells were counted per condition.

### Cell surface biotinylation

Cell surface proteins were biotinylated and isolated using the Pierce® Cell Surface Protein Isolation Kit (Thermo Fisher Scientific), following the manufacturer’s instructions. Before immunoprecipitation, the amount and concentration of protein were equalized by Lowry quantification.

### RNA extraction and quantitative reverse transcription PCR

Total RNA was isolated from cells using the RNeasy Mini Kit (Qiagen), following the manufacturer’s protocol. cDNA was reverse-transcribed using the High-Capacity cDNA Reverse Transcription Kit (Thermo Fisher). SIVA-1 mRNA expression was measured with SYBR green (Applied Biosystems) and normalized against L27 using the following primers: SIVA-1 forward: 5′-GATCACATATCGAGCGAAGA-3′, reverse: 5′-GCCTTCCCATCCACAGATCT-3′; and L27: forward: 5′-AGCTGTCATCGTGAAGAA-3′, reverse: 5′-CTTGGCGATCTTCTTCTTGCC-3′. Analysis was performed using the 7900HT Sequence Detection System 2.3 Software (Applied Biosystems). Relative expression fold change was determined by the comparative 2^(−ΔΔCT)^ method^21^.

### Experimental design and statistical analysis

All results were confirmed in at least three independent experiments. Representative images and western blots are shown in figures, while measurements of all repeated experiments are measured in graphs. Data sets were plotted and analyzed using GraphPad Prism v5 (GraphPad Software; La Jolla, CA, USA). Values were excluded using the analysis to identify outliers included in the software. Values are expressed as mean ± SEM. Statistical significance was set at *p* < 0.05 and was determined by two-tailed unpaired *t* test for comparisons between two groups, or by two-way ANOVA for multiple groups. Each specific test is indicated in figure legends.

## Results

### SIVA-1 interacts with FAIM-L

**Fig. 1:**
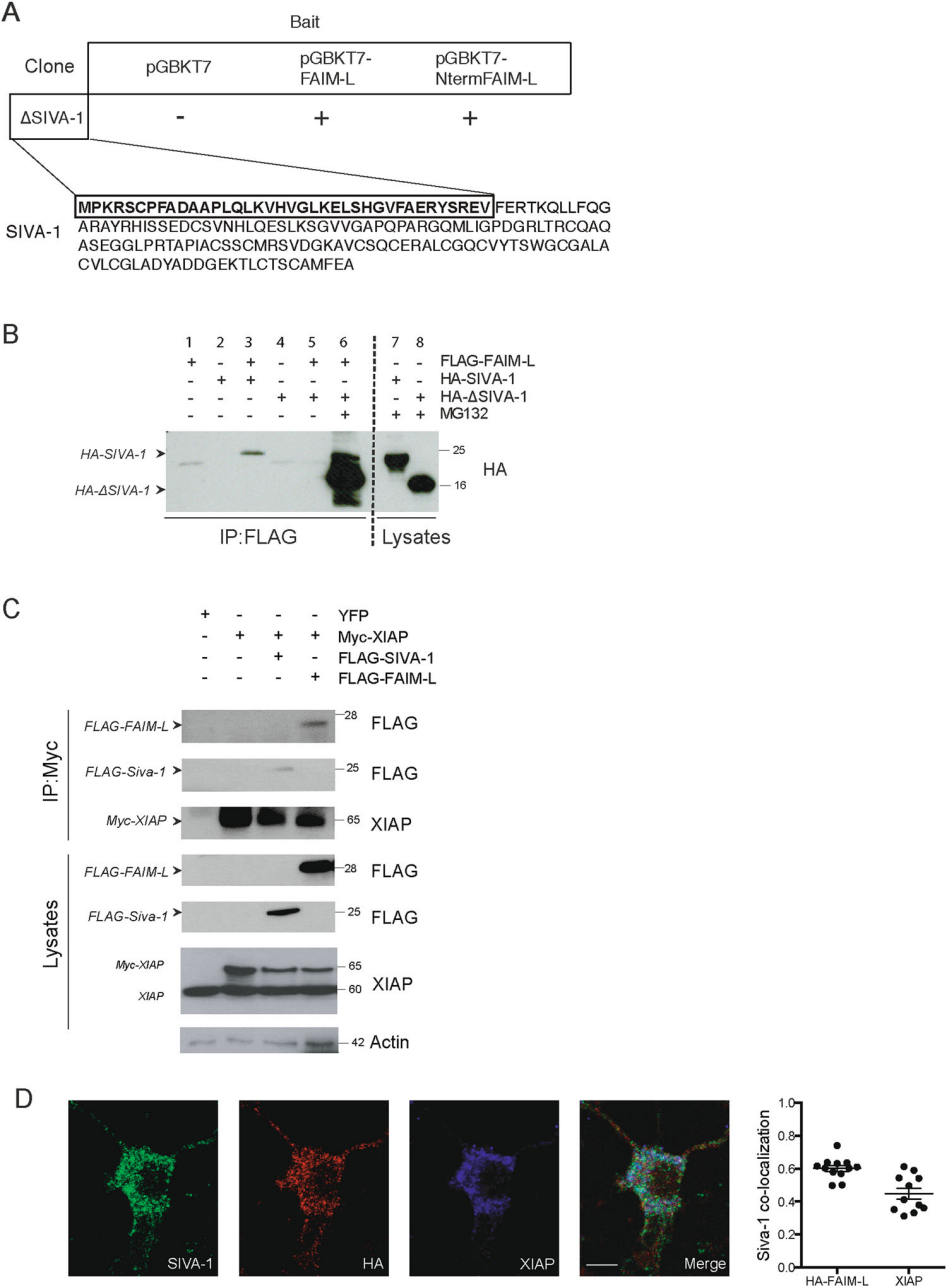
SIVA-1 interacts with FAIM-L and XIAP. **a** Schematic representation of the two-hybrid technique results. The N-terminal of SIVA-1(ΔSIVA-1) interacted with both baits, namely full-length FAIM-L and FAIM-L-specific N-terminal 22 amino acids. **b** Immunoprecipitation in HEK 293T cells of FLAG-FAIM-L using M2 Affinity FLAG. FAIM-L was immunoprecipitated and membrane was blotted with anti-HA to detect full-length SIVA-1 and ΔSIVA-1. Ten micromolar of MG132 (proteasome inhibitor) was used to impair the degradation of ΔSIVA-1. **c** Immunoprecipitation of Myc-XIAP using anti-Myc antibody. Cells were transiently transfected with Myc-XIAP, FLAG-FAIM-L, and FLAG-SIVA-1. The membrane was blotted with anti-FLAG to detect both FAIM-L and SIVA-1. XIAP was used as a control of the immunoprecipitation and actin as loading control. **d** Representative confocal images of immunocytochemistry in cortical neurons. Neurons were transfected with HA-tagged FAIM-L at 4 days in vitro. Forty-eight hour after transfection, immunofluorescence was performed staining with anti-SIVA-1 (green), anti-HA (to detect HA-FAIM-L, red), and anti-XIAP (blue). Scale bar 10 µm. Graph reports Pearson’s correlation coefficients obtained in co-localization analysis (SIVA-1/HA-FAIM-L *R* = 0.607 ± 0.060; SIVA-1/XIAP *R* = 0.412 ± 0.115).

To identify the functional partners of FAIM-L, we performed a yeast-two-hybrid screening using as bait the full-length sequence of FAIM-L or the 22 N-terminal-specific amino acids of this isoform. Among the top six hits shown in Table 1, the N-terminal fragment of SIVA-1 was detected (ΔSIVA-1) (Table 1 and Fig. 1a).

**Table 1:** Yeast two-hybrid analysis. List of protein–protein interactions with full-length FAIM-L and the 22 N-terminal-specific amino acids of the FAIM-L isoform.

Yeast two-hybrid analysis	Bait			Sequence
	pGBKT7 (control)	pGBKT7- full length FAIM-L	pGBKT7-N-term FAIM-L -(MASGDDSPIFEDDESPPYSLEK)	
SIVA-1 (ΔSIVA-1)	−	+	+	MPKRSCPFADAAPLQLKVHVGLKELSHGVFAERYSREV
FERM RhoGEF	−	+	+	HELKKDLIGIDNLVTPGREFIRLGSLSKLSGKGLQQRMFFLFNDVLLYTSRGLTASNQFKVHGQLPLYGMTIEESEEEWGVPHCLTLRGQRQSIIVAASSRSEMEKWMEDIQMAIDLAEKSNGPTPELLASSPPDNKSPDEA
GAPDH	−	+	+	YSNRMVDLMAYMASKE
COMMD1	−	+	+	EGGKSLSGLLSGLAQNAFHGHSGVTEELLHSQLYPEVPPEEFRPFLAKMRGLLKSIASADMDFNQLEAFLTAQTKKQGGITSEQAAVISKFWKSHKIKIRESLMKQSRWDNGLRGLSWRVDGKSQSRHSTQIHSPVAIIELEFGKNGQESEFLCLEFDEVKVKQILKKLSEVEESINRLMQAA
Calcyclin	−	+	+	DKFVKIYITLTGVHQVPTENVQVHFTERSFDLLVKNLNGKNYSMIVNNLLKPISVESSSKKVKTDTVIILCRKKAENTRWDYLTQVEKECKEKE
AP2m1	−	+	+	PKRACQFNRTQLGDCSGIGDPTHYGYSTGQPCVFIKMNRVINFYAGANQSMNVTCVGKRDEDAENLGHFVMFPANGSIDLMYFPYYGKKFHVNYTQPLVAVKFLNVTPNVEVNVECRINAANIATDDERDKFAGRVAFKLRINKT

We validated this interaction in HEK 293T cells by co-immunoprecipitation experiments using full-length FLAG-tagged FAIM-L, HA-tagged N-terminal SIVA-1 (ΔSIVA-1), or HA-tagged full-length SIVA-1. We confirmed the interactions between FAIM-L and both full-length and ΔSIVA-1 forms (Fig. 1b, lanes 3 and 6). Interaction with the truncated form of SIVA-1 was detected only in the presence of the proteasome inhibitor MG132 (Fig. 1b, lane 6), otherwise it was not stable and was consequently degraded.

Our group reported XIAP as a FAIM-L-interacting partner^10^. XIAP is an inhibitor of caspases and its endogenous levels are crucial for FAIM-L protection against caspase activation and consequent apoptotic or non-apoptotic functions^10,11^. Since Resch and colleagues described an interaction between SIVA-1 and XIAP^13^, we performed co-immunoprecipitation experiments and corroborated that XIAP does indeed interact with both FAIM-L and SIVA-1 (Fig. 1c).

To detect the interaction in a semi-endogenous condition, we transfected primary cortical neurons with 3xHA-FAIM-L. We then performed immunocytochemistry against HA, endogenous SIVA-1 and XIAP (Fig. 1d). Co-localization analysis revealed partial correlation of signals of SIVA-1 with both XIAP (Pearson’s correlation value *R* = 0.412 ± 0.115) and HA-FAIM-L (Pearson’s correlation *R* = 0.607 ± 0.060). We could also observe that the interactions between SIVA-1, XIAP and tagged FAIM-L takes place in neuronal cytoplasm.

### SIVA-1 is expressed in neurons

**Fig. 2:**
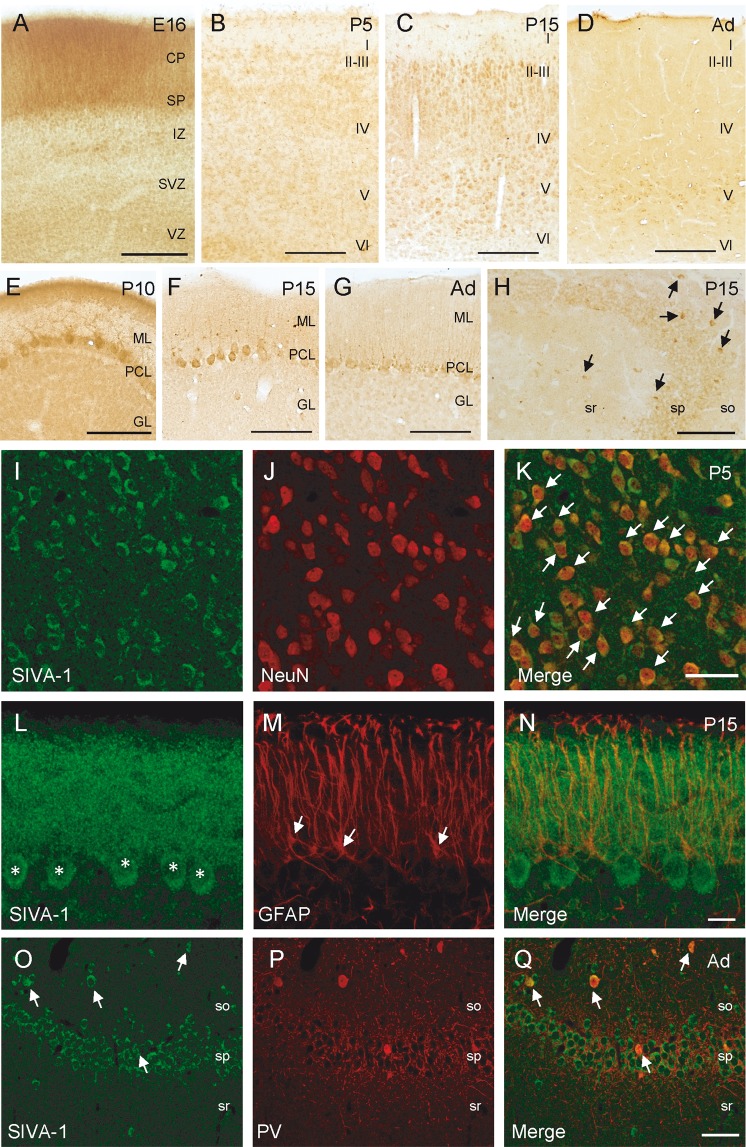
SIVA-1 is expressed in neurons during the development of the mouse brain. **a** At embryonic day 16 (E16), neurons in the cortical plate (CP) and subplate (SP) contained SIVA-1. Scale bar 100 μm. **b** At postnatal day 5 (P5), SIVA-1-immunopositive cells were located throughout all cortical layers. Scale bar 200 μm. **c** At postnatal day 15 (P15), SIVA-1-immunoreactive cells were present in all cortical layers. Note that cells located in layers II–III showed intense SIVA-1-immunolabeling. Scale bar 200 μm. **d** In the cerebral cortex of adult mice, expression of SIVA-1 was restricted to cortical cells in layer V. Scale bar 200 μm. **e**–**g** In the cerebellum, the soma and dendrites of Purkinje cells showed SIVA-1 immunostaining at all postnatal stages analyzed. Scale bar 200 μm. **h** In the hippocampus, pyramidal neurons and hippocampal interneurons expressed SIVA-1. Some hippocampal interneurons scattered in all hippocampal layers showed a strong immunostain (arrows in (**h**)). Scale bar 200 μm. **i**–**k** Double immunofluorescence of SIVA-1 and NeuN (arrows) in cortical layer V at P5, showing an almost complete co-localization of the two markers. Scale bar 25 μm. **l**–**n** Immunofluorescence co-localization of SIVA-1 and GFAP in cerebellum at P15 shows SIVA-1 expression in Purkinje cells (asterisks) but not in astrocytes (arrowheads) in the cerebellum. Scale bar 25 μm. **o**–**q** Immunofluorescence co-localization of SIVA-1 and PV shows expression of SIVA-1 in hippocampal interneurons (arrows). MZ marginal zone, IZ intermediate zone, SVZ subventricular zone, VZ ventricular zone, so stratum orines sp stratum pyramidale, sr stratum radiatum, ML molecular layer, PCL Purkinje cell layer, GL granule cell layer.

FAIM-L expression is restricted to neurons^8^. To understand the biological context in which SIVA-1 may modulate FAIM-L functions, we first examined SIVA-1 expression in mouse brain tissue. Histological analysis showed that SIVA-1 expression occurred in most brain regions throughout development (Fig. 2a–h, stages E16-adult mouse). High levels were detected in the telencephalon (cerebral cortex and hippocampus) and in the cerebellum (Fig. 2e–h). In the embryonic cerebral cortex, labeled cells were located in the cortical plate (CP) and in the subplate (SP), corresponding to postmitotic neurons of these layers (Fig. 2a). At postnatal stages (P0-P15), SIVA-1-positive cells were found throughout the cortical layers, the strongest staining being detected in layers II–III (Fig. 2b, c). At adult stages, the expression of SIVA-1 in the cerebral cortex appeared to be restricted to layer V cortical neurons (Fig. 2d). The cerebellum also showed intense SIVA-1-immunolabeling (Fig. 2e–g). SIVA-1 was expressed in migratory Purkinje cells in the embryonic cerebellum (data not shown). At postnatal P10 and P15 stages, cell bodies and dendrites of Purkinje neurons showed intense SIVA-1-immunostaining (Fig. 2e). These neurons maintained SIVA-1 expression at later stages and also during adulthood (Fig. 2g). At embryonic stages, cells in the hippocampal plate expressed SIVA-1 (data not shown). At postnatal stages, pyramidal neurons in CA1–3 and some interneurons throughout the hippocampal layers showed SIVA-1-immunostaining (Fig. 2h, o–q). To confirm the localization of SIVA-1 in neurons, P5 and P15 sections were double immuno-labeled with SIVA-1 and the neuron-specific marker NeuN. This approach revealed an almost complete co-localization of both markers at all the developmental stages analyzed (Fig. 2i–k). Double immunofluorescent studies with the astroglial cell marker Glial fibrillary acidic protein (GFAP) did not show co-localization with SIVA-1 (Fig. 2l–n). Finally, by double immunodetection of SIVA-1 with a specific interneuron subtype marker, parvalbumin (PV), we confirmed the presence of SIVA-1 in hippocampal neurons (Fig. 2o–q). We concluded that SIVA-1 protein is predominantly expressed in developing and adult neurons.

**Fig. 3:**
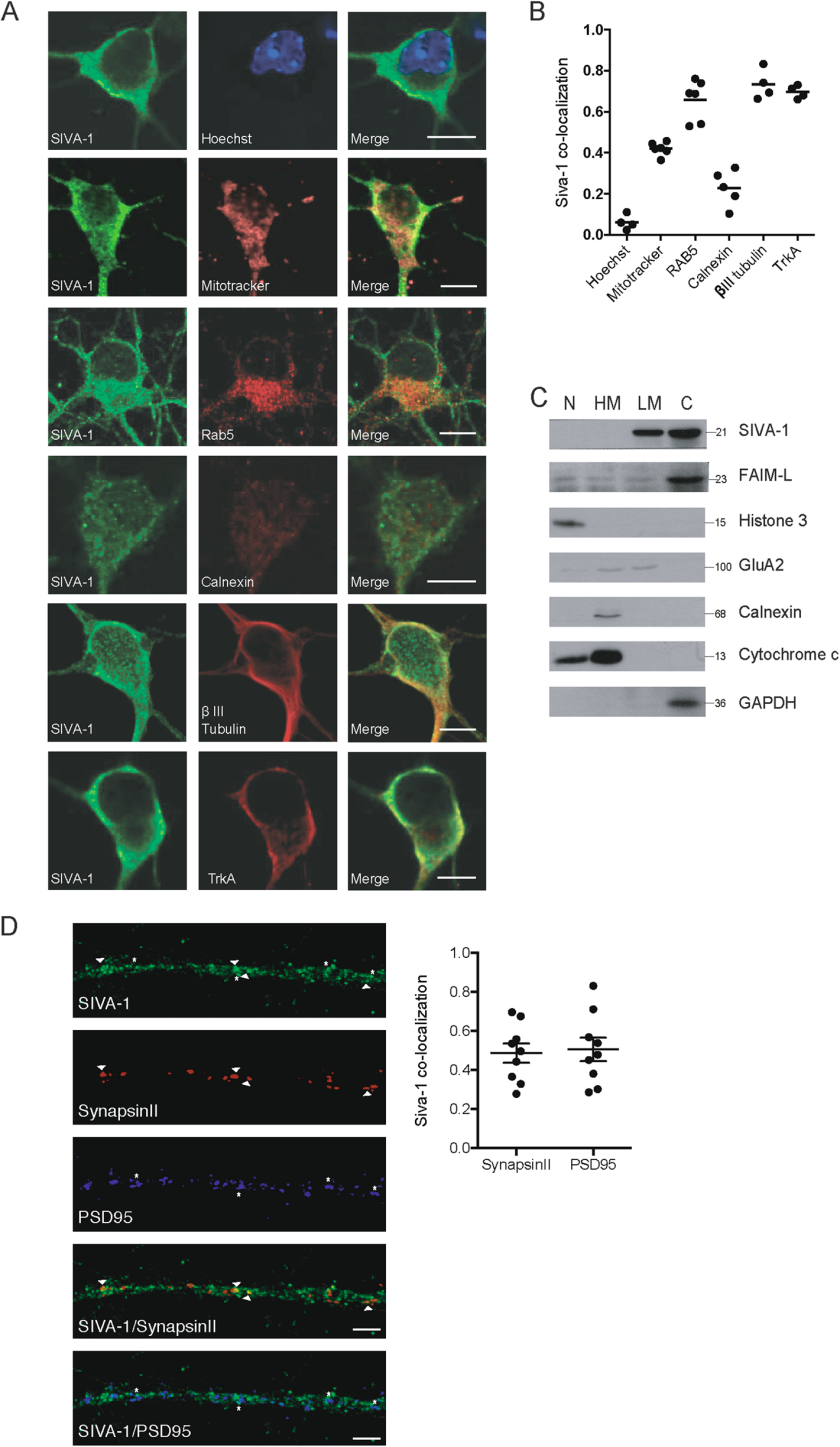
SIVA-1 has a mainly cytosolic distribution in neurons. **a** Representative confocal images of hippocampal neurons. At 7 days in vitro, immunofluorescence was performed by staining with anti-SIVA-1 (green), Hoechst (nucleus marker, blue). MitoTracker (mitochondria marker), anti-Rab5 (early endosome marker), anti-calnexin (ER marker), anti-β III Tubulin (cytoskeleton marker), and anti-TrkA (cytosolic membrane marker) were stained in red. Scale bar 10 µm. **b** Graph represents Mander’s coefficients M1 corresponding to the channel 1 (SIVA-1) signal that co-located with the channel 2 (cellular markers) signal. Only values higher than 0.5 were considered to indicate co-localization. **c** Subcellular protein fractionation assay of adult mouse brain. Histone 3 antibody was used as a nuclear marker, GluA2 as a plasma membrane marker, anti-calnexin as reticular membrane protein, cytochrome C as a mitochondrial protein, and GAPDH as a cytosolic protein. N nuclear fraction, HM heavy membrane fraction, LM light membrane fraction, C cytosolic fraction. **d** Representative confocal images of hippocampal neurites. At 14 days in vitro, immunofluorescence was performed by staining with anti-SIVA-1 (green), presynaptic markers synapsin II (red) postsynaptic marker PSD95 (blue). The graph represents Mander’s coefficient corresponding to co-localization between SIVA-1 and the synaptic markers. SIVA-1 was found to locate in some of the synapses (arrows and asterisks). Scale bar 2 µm.

To determine the subcellular localization of SIVA-1, we performed immunocytochemistry in primary cultures of hippocampal neurons. We analyzed SIVA-1 distribution in the cell body, performing co-localization analysis with several organelle markers (i.e., MitoTracker for mitochondria, Rab5 for early endosome, Calnexin for endoplasmic reticulum; Fig. 3a). Manders co-localization coefficients were obtained for each marker (Fig. 3b) and only values higher than 0.5 were considered to indicate co-localization. SIVA-1 showed a mainly cytosolic distribution and co-localized with markers of the cytoplasmic membrane (TrkA; M1 = 0.68 ± 0.02), endocytic vesicles (Rab5; M1 = 0.66 ± 0.15), and cytoskeleton (βIII-tubulin; M1 0.71 ± 0.14). SIVA-1 did not localize to mitochondria (MitoTracker; M1 = 0.41 ± 0.04) or to the endoplasmic reticulum (Calnexin; M1 = 0.22 ± 0.15). SIVA-1 expression was clearly excluded from the nucleus (Hoechst staining; M1 = 0.06 ± 0.03) (Fig. 3a, b). A subcellular protein fractionation assay confirmed the localization of SIVA-1 in cytosolic and light membrane fractions. As expected FAIM-L also was found in cytosolic fraction (Fig. 3c). We also examined SIVA-1 co-localization with synaptic markers in the neurites of 14 DIV hippocampal neurons and found that SIVA-1 can also locate in presynaptic and postsynaptic terminals as indicated by a positive correlation of SIVA-1 immunostaining with both PDS95 and synapsin II (Fig. 3d).

Taken together, our data indicate that SIVA-1 is expressed in the cytoplasm of neurons during development and adult stages. Co-expression of FAIM-L and SIVA-1 in adult neurons in basal conditions suggests that the latter participates in physiological nonlethal processes.

### SIVA-1 induces XIAP ubiquitination

XIAP is a potent inhibitor of effector caspases. Its ubiquitination and degradation by the proteasome is crucial for caspase activation and induction of apoptosis^9^. FAIM-L is a XIAP-interacting protein that executes its antiapoptotic function by inhibiting XIAP ubiquitination, hence stabilizing its levels and inhibition on caspases.

Having reported SIVA-1 interaction with both proteins we proceeded to address whether SIVA-1 interferes with the FAIM-L/XIAP interaction and XIAP ubiquitination.

**Fig. 4:**
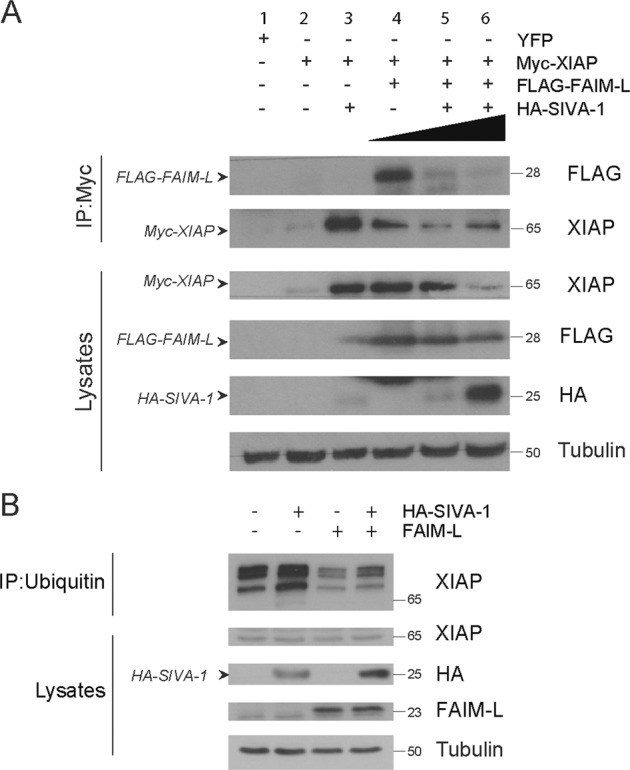
SIVA-1 promotes XIAP ubiquitination. **a** PC12 cells were transiently transfected with Myc-XIAP, FLAG-FAIM-L, and two concentrations of HA-SIVA-1. Myc-XIAP was immunoprecipitated using anti-Myc antibody. The membrane was blotted with anti-FLAG to detect FAIM-L and anti-HA to detect SIVA-1. The antibody against XIAP was used as a control of the immunoprecipitation and anti-tubulin as loading control. **b** Cells were transfected with FAIM-L and SIVA-1 (HA-SIVA-1), and endogenous ubiquitin was immunoprecipitated using anti-ubiquitin antibody. The membrane was blotted with FAIM-L, XIAP, and HA to detect SIVA-1, and tubulin was used as a loading control.

To this end, we analyzed the FAIM-L/XIAP interaction in the presence of increasing amounts of overexpressed SIVA-1 in PC12 cells (Fig. 4a, lanes 4–6). We performed an immunoprecipitation of Myc-XIAP and found the amount of co-immunoprecipitated FAIM-L to be inversely correlated to SIVA-1 overexpression. Our results thus suggest that SIVA-1 reduces the interaction of FAIM-L with XIAP.

Figure 4b shows ubiquitin immunoprecipitation in HEK293T cells overexpressing HA-tagged SIVA-1, FAIM-L, or a combination of both proteins. XIAP presented basal endogenous ubiquitination, which was enhanced in the presence of SIVA-1 overexpression. FAIM-L overexpression on the other hand reduced XIAP ubiquitination levels, thereby confirming our previous findings^10^. Moreover, overexpression of FAIM-L partially restored SIVA-1-induced XIAP ubiquitination. Overall, our results suggest that SIVA-1 and FAIM-L exert opposite effects on XIAP ubiquitination levels.

### SIVA-1 overexpression induces neuronal death by caspase-3 activation

SIVA-1 was first reported as a proapoptotic protein, and its overexpression has been shown to induce apoptosis in various cell lines^12,22–24^.

Here, we explored the effects of ectopic expression of SIVA-1 in primary hippocampal neurons.

**Fig. 5:**
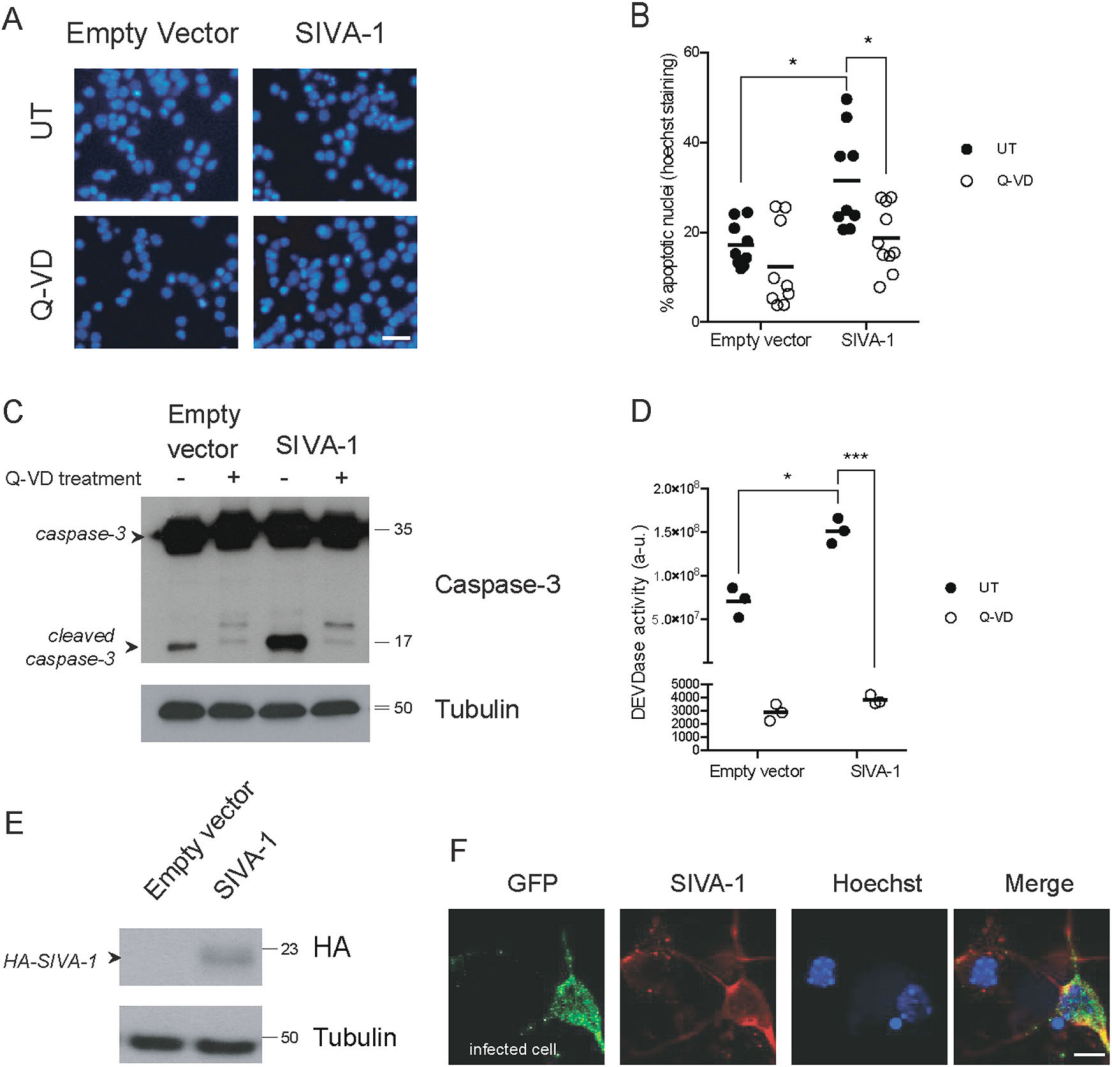
SIVA-1 induces caspase-3-dependent cell death in neurons. Primary hippocampal neurons were infected for 72 h with lentiviral particles carrying HA-SIVA-1 plasmid. **a** Representative images of Hoechst staining in control and overexpressing cells in the absence or presence of Q-VD, scale bar 20 µm. **b** Quantification of the percentage of nuclei with apoptotic morphology by Hoechst staining. SIVA-1 induced apoptosis, Q-VD treatment rescued cells from SIVA-1-induced cell death. Two-way ANOVA (empty vector vs. SIVA-1, *F* (1, 36) = 8.537 in untreated condition; *F* (1, 36) = 7.787 in Q-VD condition) *p ≤ 0.05. **c** Treatment with 10 µM of pan-caspase inhibitor Q-VD abrogated caspase-3 cleavage. The membrane was immunoblotted with anti-caspase-3 and anti-tubulin as a loading control. **d** DEVDase activity was measured in cells overexpressing SIVA-1 or control empty vector. SIVA-1 induced the activation of caspase-3 (*F* (1, 8) = 38.69). Q-VD treatment abrogated caspase-3 activity (*F* (1, 8) = 289.9) UT untreated, Q-VD quinolyl-Val-Asp-OPh. Two-way ANOVA *p ≤ 0.05; ***p ≤ 0.001. **e** SIVA-1 expression verified by western blot. HA was used to detect SIVA-1; tubulin was used as a loading control. **f** Immunocytochemistry of SIVA-1 and GFP to confirm lentiviral transfection and overexpression. Hoechst staining was used as a nuclear marker. Scale bar 10 µm.

The overexpression of SIVA-1 was sufficient to induce the typical hallmarks of apoptosis, such as chromatin condensation and/or fragmentation, in 10% of the neurons (Fig. 5a, b) respect to the empty vector, strong cleavage of caspase-3 (Fig. 5c), and an evident increase in caspase-3 activity (Fig. 5d). Treating cells with the pan-caspase inhibitor Q-VD consistently abrogated SIVA-1-induced apoptosis (Fig. 5a, b), thereby indicating that SIVA-1 induces caspase-dependent cell death. Western blot analysis of HA-SIVA-1 (Fig. 5e) and immunocytochemistry analysis by counting GFP-positive cells (Fig. 5f) confirmed SIVA-1 overexpression by lentiviral vector in murine hippocampal neurons.

### SIVA-1 modulates GluA2 internalization in hippocampal neurons

XIAP and FAIM-L modulate the non-apoptotic function of caspases, such as AMPAR GluA2 subunit internalization, the main mechanism of chLTD^4,11,25^. We found that SIVA-1 interacts with both XIAP and FAIM-L, and is an activator of caspases. We therefore proceeded to analyze whether SIVA-1 also regulates this plasticity process.

**Fig. 6:**
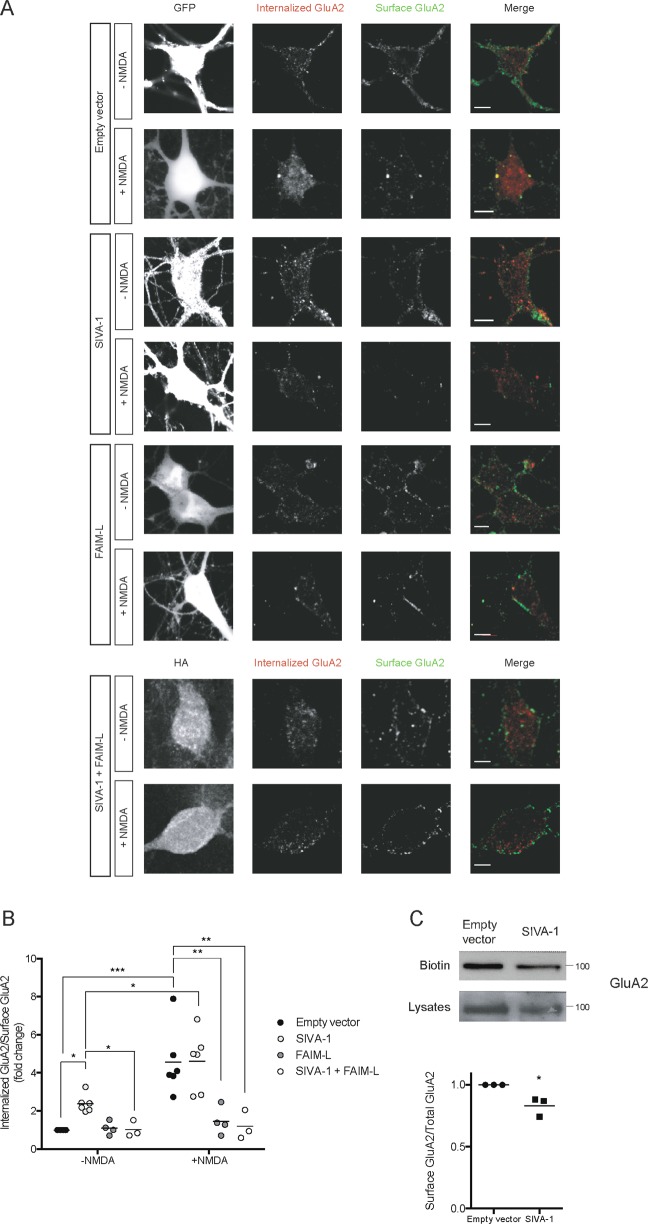
SIVA-1 promotes GluA2 internalization. **a** Representative confocal images of neurons transfected with SIVA-1, FAIM-L, or empty vector. GluA2 internalization assay was performed in neurons treated with NMDA to stimulate LTD and in untreated neurons. Only GFP-positive cells (first column) were considered for quantification. Internalized GluA2 (second column, red in merge) and surface GluA2 (third column, green in merge) were measured. Scale bar 10 µm. **b** Results were normalized to empty vector, untreated cells. Induction of chemical LTD induced GluA2 internalization in empty vector condition and SIVA-1 transfected cells (*F* (1, 632) = 15.85). Non-stimulated cells overexpressing SIVA-1 showed an increase in GluA2 internalization. FAIM-L overexpression blocked LTD induction (*F* (3, 632) = 15.17), and its overexpression with SIVA-1 in untreated cells restored basal levels of receptor internalization (*F* (3, 632) = 15.85). Each point represents an independent experimental repeat in which 15–20 cells were analyzed. UT untreated, NMDA N-methyl-D-aspartate. **c** GluA2 surface receptor was isolated with the Biotin surface assay in hippocampal cells transfected with SIVA-1 or empty vector as a control. Lower panel shows quantification of GluA2 surface receptor (*t* (2) = 3,060). Two-way ANOVA *p ≤ 0.05; **p ≤ 0.01; ***p ≤ 0.0001.

We performed a GluA2 internalization assay. Although no change was detected in SIVA-1-overexpressing neurons once NMDA treatment had been applied to induce chLTD, we found that the sole ectopic expression of SIVA-1 was sufficient to induce an increase in the internalization levels of the endogenous AMPAR subunit GluA2 (Fig. 6a–c) in neurons. SIVA-1-induced internalization was blocked by the overexpression of FAIM-L, thereby confirming a functional interaction between these two proteins. As previously described in Martinez-Marmol et al.^11^, FAIM-L overexpression abrogated GluA2 internalization induced by chLTD.

**Fig. 7:**
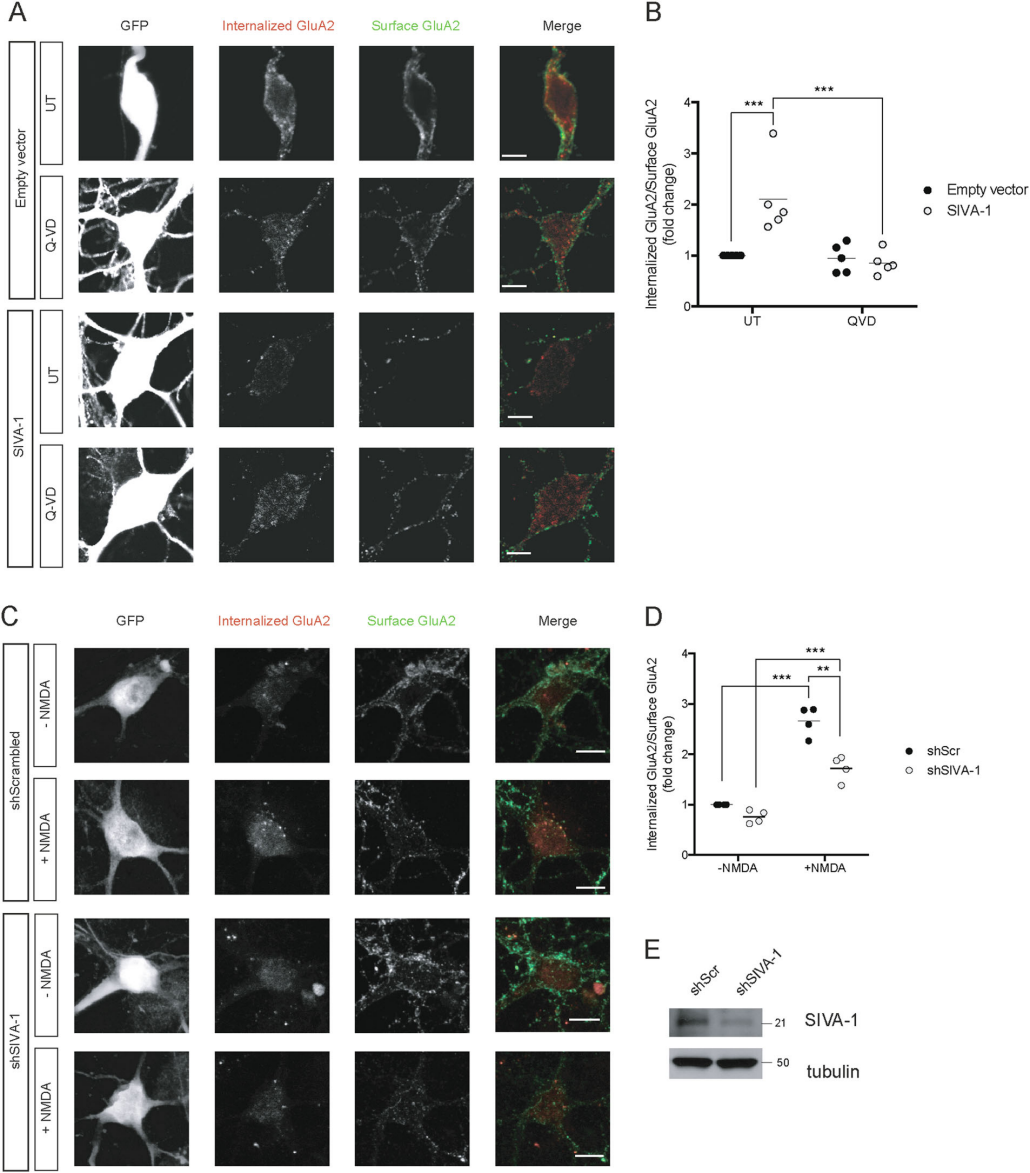
SIVA-1 modulates AMPAR internalization in a caspase-dependent manner. **a** Representative confocal images of neurons transfected with SIVA-1 or empty vector and treated with 10 μM of Q-VD (caspase inhibitor). Only GFP-positive cells (first column) were considered for quantification (left panel). Scale bar 10 µm. **b** The results of the quantification of internalized GluA2 vs. surface GluA2 were normalized to empty vector, untreated cells (right panel). Each point represents an independent experimental repeat in which 15–20 cells were analyzed. SIVA-1-transfected cells showed greater internalization of GluA2 receptor (*F* (1, 97) = 3.840), Q-VD treatment restored internalization to basal levels (*F* (1, 97) = 7.345). Two-way ANOVA ***p ≤ 0.001. **c** Representative confocal images of neurons transfected with vectors carrying shSIVA-1 or shScramble as control. The GluA2 internalization assay was performed in neurons treated with NMDA to stimulate LTD and in untreated neurons. Only GFP-positive cells (first column) were considered for quantification. Internalized GluA2 (second column, red in merge) and surface GluA2 (third column, green in merge) were measured. Scale bar 10 µm. **d** The results of the quantification of internalized GluA2 vs. surface GluA2 were normalized to shScrambled, untreated cells. Each point represents an independent experimental repeat in which 15–20 cells were analyzed. NMDA treatment induced GluA2 internalization in both shScrambled and shSIVA-1 cells (*F* (1, 84) = 167.1). However, shSIVA-1 cells treated with NMDA showed a significant decrease in receptor internalization (*F* (1, 84) = 33.5). Two-way ANOVA **p ≤ 0.01; ***p ≤ 0.001. shScr shScrambled.

Given that caspases are essential for GluA2 internalization, we addressed whether the decrease in GluA2 basal surface level in SIVA-1-overexpressing neurons was caspase-dependent. The presence of the pan-caspase inhibitor Q-VD totally abrogated SIVA-1-induced internalization of GluA2 (Fig. 7a, b), indicating that SIVA-1 modulates postsynaptic receptor levels in a caspase-dependent manner.

To further examine the role of SIVA-1 in the regulation of synaptic receptor internalization, we knocked down SIVA-1 levels and analyzed GluA2 internalization. A decrease in SIVA-1 levels was observed when a shRNA was used (Fig. 7e). Although no change was observed in untreated cells, we did detect a decrease in GluA2 internalization in shSIVA-1-infected neurons after the induction of chLTD. GluA2-induced internalization was marked in shSIVA-1 neurons, but significantly decreased compared to control infected neurons.

### SIVA-1 levels are upregulated in chLTD induction

**Fig. 8:**
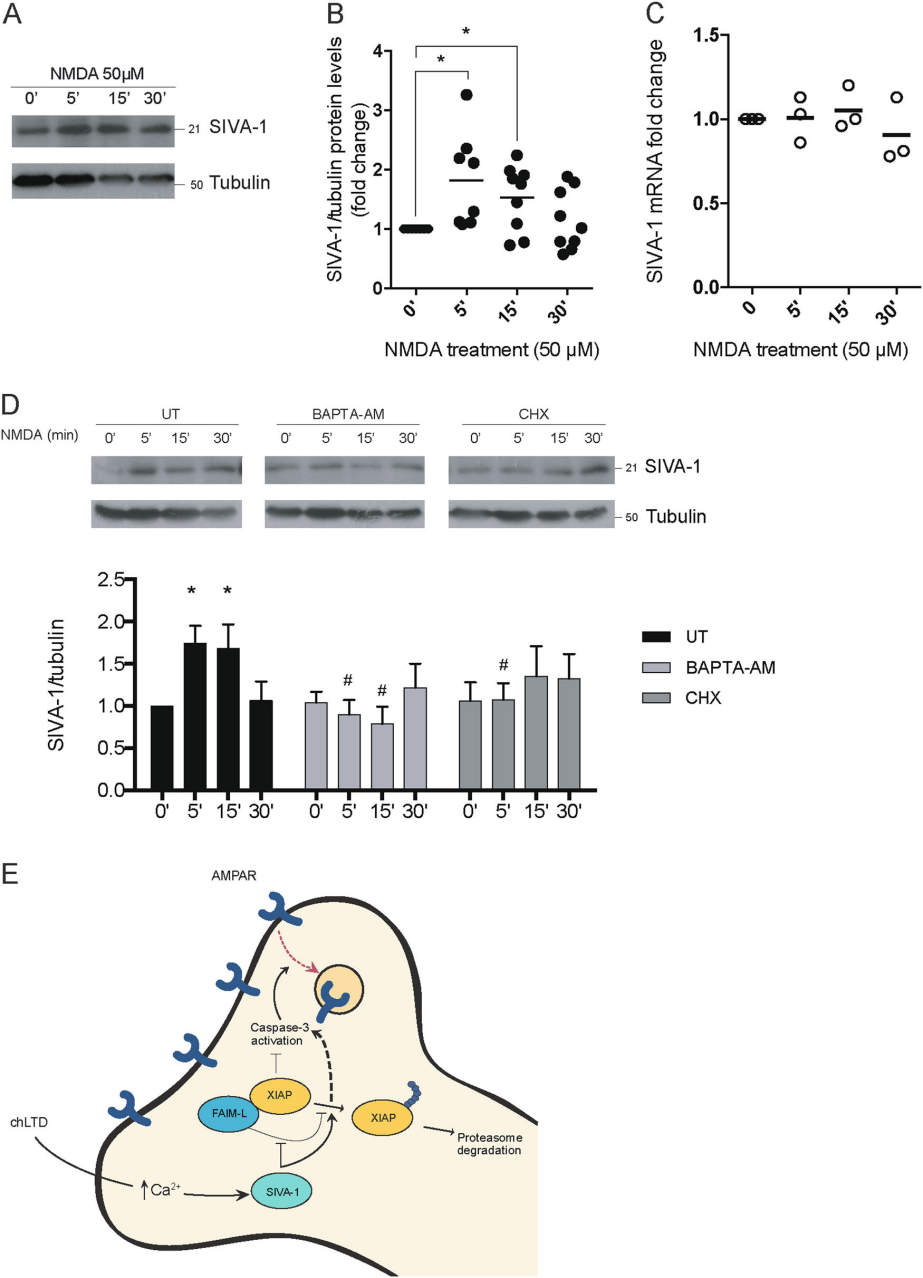
Levels of SIVA-1 increase after NMDA treatment. **a** Primary neuronal cells were treated with 50 μM of NMDA at different time points (0–30 min) and protein expression of SIVA-1 was analyzed by SDS-PAGE. Anti-tubulin was used as a loading control. **b** SIVA-1 expression increased with 5 min, and 15 min treatment of NMDA. *t* test (*t* (15) = 2427; *t*(15) = 2755). *p ≤ 0.05. **c** qPCR revealed no change in SIVA-1 mRNA expression after treatment. **d** The increase in SIVA-1 levels (two-way ANOVA *F*(2, 6) = 1712) was blocked by pretreatments with 50 µM of BAPTA-AM for 30 min or 1 µg/ml of cycloheximide for 1 h (two-way ANOVA (*F* (3, 9) = 1326)). *p ≤ 0.05 for comparison between time points in the same treatment, ^#^*p* ≤ 0.05 for comparison between treatments. **e** Schematic representation of SIVA-1 function. In neurons, SIVA-1 was increased after chemical LTD induction. SIVA-1 destabilized XIAP by increasing its ubiquitination and impairing its interaction with FAIM-L. We propose that this is one of the mechanisms through which SIVA-1 triggers caspase-3 activation in neurons and consequent apoptosis and caspase-3-dependent GluA2 internalization. CHX cycloheximide, BAPTA-AM 1,2-Bis(2-aminophenoxy)ethane-N,N,N’,N’-tetraacetic acid tetrakis(acetoxymethyl ester), chLTD chemical long-term depression.

Our findings suggest a role of SIVA-1 in caspase-dependent GluA2 internalization. The expression of several modulators of synaptic plasticity is required for the correct induction and maintenance of plasticity changes^26^. Analysis of SIVA-1 protein levels during chLTD induction showed that they were rapidly induced after 5 min of NMDA treatment (Fig. 8a, b), while SIVA-1 mRNA levels were unaltered (Fig. 8c). The inhibition of protein translation by cycloheximide blocked the increment of SIVA-1 (Fig. 8d), thereby suggesting posttranscriptional regulation.

Induction of chLTD by NMDA stimulation leads to an increase in Ca^2+^ inflow in neurons, followed by calcineurin and caspase-3 activation, and the internalization of AMPAR. Blockage of calcium by BAPTA-AM treatment inhibited the increase in SIVA-1 expression after chLTD. This observation thus suggests that the first steps of the NMDA stimulation pathways are essential for SIVA-1 induction (Fig. 8d).

In conclusion, we propose SIVA-1 as a new modulator of synaptic plasticity. We demonstrate that SIVA-1 is increased in chLTD, where it activates caspase-3. We propose that SIVA-1, by modulating the FAIM-L/XIAP interaction, induces XIAP degradation and consequent activation of caspases. SIVA-1 therefore emerges as a novel regulator of caspase-dependent apoptotic and non-apoptotic functions in neurons (Fig. 8e).

## Discussion

Apoptosis is an essential process for the homeostasis of organisms. It is subjected to multiple and tight mechanisms of regulation, but it can also be unscheduled triggered in pathological processes such as degenerative diseases. In these pathologies, the mechanisms of cellular protection (i.e., antiapoptotic mechanisms) fail. Understanding the underlying components of the dysregulation may provide new opportunities for therapeutic intervention. The disassembly of neuronal integrity during cell death is regulated by cysteine-aspartic acid proteases called caspases. These proteins have been widely characterized for their role in apoptosis^27^ and in non-apoptotic functions. In neurons, caspases locate in dendrites, axons, and in presynaptic and postsynaptic terminals. At these sites, they can be locally activated^4^ and participate in synaptic plasticity and growth cone motility, among other non-apoptotic functions^28^.

FAIM-L is a neuron-specific death receptor antagonist that protects neurons against a variety of neurotoxic insults^6,8,10,29^. We have reported that FAIM-L protects XIAP, an inhibitor of caspases, from ubiquitination and degradation. By stabilizing XIAP levels, FAIM-L regulates caspase-3 activation in apoptosis and exerts non-apoptotic functions^11^. To characterize FAIM-L in depth, we performed a two-hybrid assay to detect interacting proteins.

We identified and confirmed SIVA-1 to be a functional partner of FAIM-L. SIVA-1 was first reported as a CD27-interacting protein^12^. SIVA-1 plays a critical proapoptotic role in several cellular types and is overexpressed in various pathological circumstances, such as ischemic injury and Coxsakie virus B3 infection^14,30–32^. Resch et al.^13^ demonstrated that SIVA-1 interacts with XIAP, and suggested that the interaction leads to mutual interference with their action.

Our analysis of SIVA-1 expression and distribution in brain tissues at embryonic and adult stages shows that this protein is expressed in both developing and adult neurons. Nervous system development requires a precise regulation of programmed cell death, and many apoptotic proteins have been found to be crucial. Among these, SIVA-1 appears to play a key role during development, as its deletion has been reported to result in embryonic lethality in mice^33^. The presence of SIVA-1 expression is maintained in adult cortical and hippocampal neurons, thereby suggesting that this protein contributes to neuronal physiological functions.

We examined the subcellular distribution of SIVA-1 in primary hippocampal neurons. Previous studies carried out in various cell types reported controversial findings on the subcellular localization of this protein. Some described that the ectopic expression of SIVA-1 accumulates in the nucleus^24,34,35^ and that it can interact with cell surface membrane receptors^12,23,24,34^. Moreover, Jacobs et al.^14^ reported membrane localization of SIVA-1 upon DNA-damage induced in the brain. However, most of the functions exerted by SIVA-1 in apoptosis take place in the cytoplasm^36^. Our analysis shows that, in cultured hippocampal neurons, SIVA-1 is located in cellular bodies, neurites and also in synapses, and that it presents a diffuse cytosolic pattern that excludes the nucleus, similar to the localization pattern described for FAIM-L.

We then analyzed the possible functions of SIVA-1 in relation to the previously described FAIM-L function on XIAP modulation^10,11^. Our data show that SIVA-1 overexpression displaces the FAIM-L/XIAP interaction. Loss of interaction with FAIM-L can be sufficient to promote XIAP instability, thereby inducing ubiquitination-mediated proteasomal degradation. We reveal that SIVA-1 overexpression also has a direct effect on promoting XIAP ubiquitination. Given that SIVA-1 is a XIAP-interacting protein, it might confer structural changes to XIAP that permit auto-ubiquitination, or it may serve as an ubiquitin ligase on XIAP. In fact, SIVA-1 has been described to have E3 ubiquitin ligase activity and to direct E3 ubiquitin ligases to promote the ubiquitination of other proteins and consequent changes in their function or stability^37,38^. In a complex process such as apoptosis, which involves hundreds of molecules, the regulation between proapoptotic and antiapoptotic proteins is common and can often be mediated by ubiquitination^39^.

Here, we report the proapoptotic activity of SIVA-1 in primary cultures of hippocampal neurons. SIVA-1 overexpression is sufficient to induce apoptosis through effector caspase activation. We propose that the promotion of cell death induced by SIVA-1 overexpression occurs through XIAP destabilization and consequent inhibition of XIAP action on effector caspases. Here we provide evidence that SIVA-1 participates in XIAP modulation and apoptosis and that it exerts the opposite function to FAIM-L.

We addressed whether SIVA-1 has a neuron-specific non-apoptotic role. We demonstrate that SIVA-1 is expressed in cultured hippocampal neurons in the absence of proapoptotic stimuli. This finding points to a non-apoptotic role of the protein. We studied LTD, a well characterized caspase-dependent mechanism, which requires a controlled activation of the same mitochondrial pathway and that involves the same proteins that can lead to apoptotic cell death. Many apoptotic regulatory proteins modulate caspase activation in synaptic depression, and among these we find both FAIM-L and XIAP as inhibitors of the process. We used as a model for our study the extensively described chLTD. After NMDA stimulation, chLTD is induced. A rapid increase in calcium levels is triggered by NMDA receptor activation, followed by an involvement of calcineurin and caspase-3. The activation culminates in AMPAR internalization and therefore synaptic weakening^40^. When measuring surface AMPAR levels in our cellular primary cultures, we observed that SIVA-1 induces an increase in GluA2 internalization in non-stimulated neurons in a caspase-dependent manner. Moreover, SIVA-1 knockdown significantly decreases receptor internalization following chLTD. Therefore SIVA-1 emerges as another regulator of apoptosis that is also required for functions not related to cell death. FAIM-L overexpression abrogates GluA2 internalization following chLTD and also restores SIVA-1 induction of GluA2 internalization in hippocampal neurons.

Protein composition at synapses is modulated in response to neuronal activity, through new synthesis and removal^41,42^. We show an increase in SIVA-1 protein levels in primary cultures after a few minutes of NMDA treatment. The increase is blocked by cycloheximide and does not affect SIVA-1 mRNA levels, thereby indicating specific translational regulation. The increase in SIVA-1 could be directly associated with NMDA receptor stimulation after treatment, since a calcium chelating agent, BAPTA-AM, blocks this increase. BAPTA-AM pretreatment has been reported to block LTD induction, thus eliminating available calcium ions to start a stimulation response after NMDA or electrical application^43,44^. The enhanced SIVA-1 expression in chLTD supports the relevance of this protein in the modulation of this synaptic plasticity mechanism.

In summary, our results reveal novel key roles of SIVA-1 in neurons. We show that SIVA-1 interacts with both FAIM-L and XIAP and that it is also a caspase activator. We associate SIVA-1-induced caspase activation with neural apoptosis, which is consistent with its previously described functions. Moreover, we demonstrate a novel function of SIVA-1 as regulator of synaptic function.
